# Olfaction and kidney function in community-dwelling older adults

**DOI:** 10.1371/journal.pone.0264448

**Published:** 2022-02-25

**Authors:** Keran Wang, Zhehui Luo, Chenxi Li, Jayant M. Pinto, Eric J. Shiroma, Eleanor M. Simonsick, Honglei Chen

**Affiliations:** 1 The Department of Epidemiology and Biostatistics, College of Human Medicine, Michigan State University, East Lansing, Michigan, United States of America; 2 Section of Otolaryngology-Head and Neck Surgery, Department of Surgery, The University of Chicago Medicine and Biological Sciences, Chicago, Illinois, United States of America; 3 Laboratory of Epidemiology and Population Science, Intramural Research Program of the National Institutes of Health, National Institute on Aging, Bethesda, Maryland, United States of America; Universita degli Studi Magna Graecia di Catanzaro, ITALY

## Abstract

**Background:**

In older adults, kidney function declines with age. People with advanced kidney diseases may have poor olfaction. However, it is unclear whether poor olfaction is a marker for declining renal function or future risk of chronic kidney disease (CKD). We therefore investigated olfaction in relation to kidney function and risk of CKD.

**Methods:**

These secondary data analyses were limited to participants of the year 3 clinical visit of the Health Aging and Body Composition Study. The analytic sample size varied between 1427 to 2531, depending on participant eligibility and data availability for each analysis. Olfaction was tested using the Brief Smell Identification Test (B-SIT), defined as anosmia (score≤6), hyposmia (7–8), moderate (9–10), and good function (10–11) at baseline. We estimated glomerular filter rate (eGFR) at baseline and seven years later using the CKD-EPI creatinine-cystatin C equation, and defined incident CKD as eGFR<60 ml/min/1.73m^2^ and eGFR decline ≥1 ml/min/1.73m^2^/year. Further, we identified CKD hospitalization events from hospitalization and death records. We used inverse probability weighting and weighted multivariable regressions to account for censoring in the prospective analyses and used absolute risk regression to account for competing risk of death.

**Results:**

At baseline, compared to participants with good olfaction, the multivariable-adjusted mean eGFR was 3.00 ml/min/1.73m^2^ lower (95% confidence interval (CI): -5.25, -0.75) for those with anosmia and 1.87 lower (95% CI: -3.94, 0.21) for those with hyposmia with a *P* for linear trend < 0.001. Those with anosmia at baseline was had a significantly lower eGFR seven years later (-5.31, 95% CI: -8.58, -2.04, *P* for trend = 0.002), but the association was attenuated after further accounting for baseline eGFR (-2.37, 95%CI: -4.91, 0.16, P for linear trend = 0.147). Olfactory function was not associated with incident CKD or CKD hospitalization.

**Conclusion:**

In older adults > age 70 years, poor olfaction is associated with lower kidney function, but not future CKD risk. These associations should be further investigated in relatively younger population.

## Background

A healthy kidney is critical for maintaining body homeostasis by filtering out waste and balancing body fluid levels. Human kidney function decreases with age, and its abnormal functional decline may eventually progress to chronic kidney disease (CKD) [1]. CKD is associated with premature death [2], and in the case of end-stage renal disease (ESRD), it results in substantial need for costly care and treatment [3, 4]. Such burdens are preventable if abnormal decline in kidney function or CKD can be recognized at an early stage. However, in older adults, CKD is often asymptomatic at early stages and requires repeated blood or urine tests of kidney function for diagnosis [2, 3]. There is a need for easy and novel ways to identify kidney function decline and CKD at their earliest stages in older adults.

The human sense of smell also declines with age, affecting up to 25% of older adults overall and >60% for those ages ≥80 [5]. Several lines of evidence suggest a potential link between poor olfaction and renal dysfunction in older adults. First, several small hospital-based case-control studies [6–14] report that patients with ESRD or CKD have worse olfaction than controls, which may be due to the toxic effects of high circulating levels of uremic waste on the regeneration of nasal olfaction epithelia [12, 15]. However, to our knowledge, no study has investigated whether poor olfaction presents in early stages of CKD or renal dysfunction. Second, it has been widely speculated that poor olfaction may gradually diminish the appetite of those affected, degrade their food choices, and result in weight loss and malnutrition [16, 17]. These may initiate or accelerate renal dysfunction, leading to CKD and poor prognosis [18]. Either as a marker of renal damage or risk factor/accelerator of CKD in older adults, an olfaction test may provide a simple and facile way to identify CKD at its earliest stages. We therefore explored olfaction in relation to current and future kidney function as well as to CKD risk in a community-dwelling cohort of older US adults.

## Methods

### Study population

The Health, Aging and Body Composition (Health ABC) Study was designed to investigate the interrelationships among health conditions, social and behavioral factors and functional changes in community-resident older adults [19, 20]. Briefly, in 1997 to 1998, the study recruited 3075 well-functioning, community-dwelling older adults aged 70–79 years (48.8% men and 41.6% blacks). Potential participants were identified from a random sample of Medicare beneficiaries for white adults and from all community-dwelling residents with eligible age in specified zip code areas surrounding the Pittsburgh, Pennsylvania, and Memphis, Tennessee for black adults. Eligibility criteria included free of reported difficulty walking ¼ mile or climbing up 10 steps, no active life-threatening cancer in the last 3 years, and no plan to move away from the study areas in 3 years. Participants completed a comprehensive clinical visit at enrollment (year 1) and were subsequently followed with similar clinical visits annually through year 6, and then in years 8, 10, 11, and 16. The study protocol of the Health ABC study was approved by all relevant institutional review boards and all participants provided written consent at enrollment.

### The Brief Smell Identification Test (B-SIT)

At the year 3 clinical visit, participants took the B-SIT, a widely used screening test for identifying olfactory deficit [21]. The B-SIT is a shorter version of the University of Pennsylvania Smell Identification Test (UPSIT) and was validated for use in older adults with various backgrounds [22]. This test asks participants to smell 12 common odorants, one at a time, and to select the correct odorant from 4 possible answers in a forced multiple-choice format. Test score consist of the number of correctly identified odorants, ranging from 0 to 12. We defined anosmia as a B-SIT score of 0–6, hyposmia as 7–8, moderate olfactory function as 9–10, and good olfactory function as 11–12, consistent with other studies [23, 24].

### Outcomes

Kidney function was assessed at years 3 and 10 by the estimated glomerular filtration rate (eGFR) according to the well-established CKD-EPI creatinine-cystatin C equation [25]. Serum creatinine was assayed at the Laboratory for Clinical Biochemistry Research(LCBR), University of Vermont, using the Kodak Ektachem 700 Analyzer (Rochester, NY) with a colorimetric assay [26, 27]. The calibration of the assay was performed using isotope dilution mass spectrometry (IDMS) as a reference for more accurate eGFR results [28]. Among the 5% blind duplicates, the coefficient of variation (CV) of paired results was 9.9% [29]. Serum cystatin C was measured at LCBR by using the BNII nephelometer (Dade Behring Inc. Deerfield, IL) utilizing a particle enhanced immunenepholometric assay (N Latex Cystatin C) [30] with a 7.8% CV in the 5% blind duplicates [29].

We assessed the CKD outcomes in two ways. In the first analysis, we defined incident CKD as eGFR<60 ml/min/1.73m^2^ at year 10 but not at year 3 and with an annual decrease of eGFR ≥1 ml/min/1.73m^2^ [31]. While this approach used eGFR measures to identify incident CKD, we had eGFR measures only at two-time points and 21.7% of study participants were died or lost to follow-up at the year 10 clinical visit. In the second analysis, we defined the analytic outcome as hospitalizations due to CKD. The Health ABC study conducted extensive hospitalization and vital surveillance throughout the follow-up. Briefly, all participants were asked to report hospitalizations at clinical visits and interim semi-annual phone interviews. For each hospitalization, up to 20 diagnoses were coded using the International Classification of Diseases, ninth edition (ICD-9). We defined CKD hospitalization event as any hospitalization with CKD (ICD-9: 585) and deaths with renal failure as a contributing or immediate cause of death.

### Covariates

Baseline sociodemographic characteristics (age, sex, race, study site, and education), lifestyle (smoking status, alcohol consumption, brisk walking), and overall health status were self-reported as part of the clinical visit. We calculated body mass index (BMI) by dividing weight in kilograms by height in meters squared (kg/m^2^). We defined diseases according to published protocols, briefly 1) diabetes as self-reports of physician-made diagnosis of diabetes, the use of anti-diabetic drugs, a fasting blood glucose level of at least 126mg/dL, or an oral glucose tolerance test result of 200mg/dL or higher [32]; 2) hypertension as self-reports of physician-made diagnosis with the use of antihypertensive drugs, or measured systolic blood pressure of at least 140 mmHg or diastolic blood pressure of at least 90 mmHg [33]; 3) cardiovascular diseases as adjudicated diagnoses of congestive heart failure, myocardial infarction, angina, cerebrovascular disease (stroke, transient ischemic attack, or carotid artery disease) and peripheral arterial disease [34]; and chronic lung diseases as asthma, bronchitis, chronic obstructive pulmonary diseases, and emphysema, adjudicated from hospitalization records [35]. Use of statins and antihypertensive drugs were assessed using the medication inventory methods, using definitions consistent with previous reports [36, 37]. Finally, total cholesterol (TC), HDL-cholesterol (HDL-C) and triglycerides were measured with fasting EDTA plasma at enrollment, and LDL-cholesterol (LDL-C) was estimated with the Friedewald equation [38]. The inter-assay CVs for TC, HDL-C, and triglyceride were 1.5%, 2.3% and 2.3% respectively at the LCBR [39]. Serum albumin level at enrollment was measured using the biomocresol green method (Vitros; Orthoclinical Diagnostics Inc., Rochester, NY, USA) [40] with an inter-assay CV for albumin of 2.0% [41].

### Statistical analysis

We conducted four analyses to comprehensively assess the association of poor olfaction with kidney function and CKD. The sample sizes for these analyses however varied due to different participant eligibility, data availability, loss to follow-up, and deaths (S1 Fig). These analyses are 1) cross-sectional analysis on olfaction and kidney function using data from the year 3 clinical visit (n = 2161); 2) prospective analysis on olfaction at year 3 in relation to the change in kidney function between year 3 and year 10 (n = 1693), 3) prospective analysis on olfaction at year 3 and incident CKD between year 3 and year 10 (n = 1427), and 4) prospective analysis on olfaction at year 3 in relation to the first CKD hospitalization (n = 2531) through year 15. Further, as described below, for each analysis, we chose different analytic strategies that could best serve the specific analytic goal.

In univariable descriptive analyses, we used the ANOVA/Kruskal-Wallis test to compare group means or distributions of continuous variables and the Chi-square test to compare proportions for categorical variables. In multivariable cross-sectional association analyses, we first assessed olfaction and kidney function at year 3 among the 2161 eligible participants (S1 Fig). We used multivariable linear regression models, first adjusting for demographic and lifestyle covariates, and then additionally for other health-related covariates as defined above. We then assessed olfaction at year 3 prospectively in relation to kidney function at year 10 among 1693 participants (S1 Fig). We conducted the multivariable analyses with and without further adjusting for year 3 eGFR and accounted for lost to follow-up using inverse probability weighting (IPW) and weighted linear regression. We calculated stabilized weights [42] for each participant for which the numerator was the probability of censoring conditional on olfaction and the denominator was the probability of censoring conditional on olfaction, covariates, and their two-way interaction terms. We used the Hosmer-Lemeshow test to assess the goodness of fit of the IPW weight model. We tested *P* for linear trend by using the median score of each category as a continuous variable. We further limited both analyses to those with baseline eGFR>60 ml/min/1.73 m^2^ to minimize the potential impacts of those with poor kidney function on the analyses.

For the analysis of incident CKD between year-3 and 10 clinical visits, we limited to participants with normal kidney function (eGFR ≥ 60 ml/min/1.73m^2^) at year 3 (n = 1427, S1 Fig). Because we do not have data on the time of CKD onset or diagnosis, we conducted the analyses using weighted logistic regression and reported odds ratio (OR) and 95% CI, accounting for loss to follow-up and potential confounders mentioned above. In the last analysis, we examined olfaction in relation to the date of CKD hospitalization among 2531 participants who had no hospitalization for CKD before year 3 (S1 Fig). In this time-to-event analysis, we chose the absolute risk regression (AAR) over Cox regression because ARR does not need to make any proportionality assumptions and it estimates the cumulative incidence ratio (CIR), accounting for competing risk of death [43]. Missing values for continuous covariates were imputed with mean, and for categorical covariates were as the category with the greatest proportion. Participants were followed from year 3 clinical visit to the date of CKD hospitalization, death, last contact, or August 14, 2012, whichever occurred first. We used R software (version 4.0.3) and the “timereg” package to conduct the time-to-event analysis; all other analyses were conducted using the SAS software (version 9.4; SAS Institute, Cary, NC) with two-sided alpha of 0.05.

## Results

At the year 3 clinic visit, eligible participants were on average 75.6 ±2.9 years old and included 52.0% females and 38.1% blacks. Using the cutoff of eGFR<60 ml/min/1.73m^2^, 19.2% of our study participants had poor kidney function. Compared to participants with good olfaction, those with hyposmia or anosmia were older, and more likely to be male, black, from Memphis, current smokers, and former drinkers (Table 1). They were also more likely to report less than high school education, antihypertensive drug uses, fair-to-poor health, and a history of diabetes, chronic lung diseases, low HDL-C, and poor kidney function.

**Table 1:** Population characteristics by baseline olfaction status (n = 2161).

Variable	Olfaction status				*P*
	Anosmia (n = 323)	Hyposmia (n = 384)	Moderate (n = 738)	Good (n = 716)	
Age in years, mean (SD)	76.4 (2.9)	75.8 (2.9)	75.6 (3.0)	75.2 (2.6)	<0.001
Male sex, n (%)	208(64.4)	218(56.8)	345(46.7)	267(37.3)	<0.001
Black race, n (%)	164(50.8)	154(40.1)	289(39.2)	217(30.3)	<0.001
Site, n (%)					
Memphis	164(50.8)	210(54.7)	372(50.4)	317(44.3)	0.006
Pittsburgh	159(49.2)	174(45.3)	366(49.6)	399(55.7)	
Education, n (%)					
<high school	112(34.7)	115(29.9)	160(21.7)	106(14.8)	<0.001
≥high school	211(65.3)	269(70.1)	578(78.3)	610(85.2)	
BMI, n (%)					
<25 kg/m^2^	116(35.9)	148(38.5)	236(32)	232(32.4)	0.110
25–30 kg/m^2^	143 (44.3)	151 (39.3)	307 (41.6)	313 (43.7)	
>30 kg/m^2^	64(19.8)	85(22.1)	195(26.4)	171(23.9)	
Smoking, n (%)					
Current	30(9.3)	34(8.9)	55(7.5)	34(4.7)	0.011
Former	165(51.1)	180(46.9)	363(49.2)	329(45.9)	
Never	128(39.6)	170(44.3)	320(43.4)	353(49.3)	
Alcohol consumption, n (%)					
Current	154(47.7)	193(50.3)	372(50.4)	394(55)	<0.001
Former	96(29.7)	90(23.4)	151(20.5)	119(16.6)	
Never	73(22.6)	101(26.3)	215(29.1)	203(28.4)	
Brisk walking, n (%)					
<90 min/wk	299(92.6)	344(89.6)	668(90.5)	626(87.4)	0.061
≥90 min/wk	24(7.4)	40(10.4)	70(9.5)	90(12.6)	
General health status, n (%)					
Fair to poor	80(24.8)	82(21.4)	113(15.3)	104(14.5)	<0.001
Excellent to good	243(75.2)	302(78.6)	625(84.7)	612(85.5)	
Diabetes, n (%)	90(27.9)	85(22.1)	167(22.6)	141(19.7)	0.035
Hypertension, n (%)	235(72.8)	284(74)	550(74.5)	509(71.1)	0.496
CVD, n (%)	83(25.7)	123(32)	217(29.4)	203(28.4)	0.304
Chronic lung diseases, n (%)	61(18.9)	75(19.5)	147(19.9)	100(14)	0.015
LDL-C in mg/dL, mean (SD)	121.2 (35.4)	120.8 (34.8)	120.9 (32.4)	122.5 (36.5)	0.807
HDL-C in mg/dL, median (IQR)	40.0 (11.0)	42.0 (11.0)	41.0 (11.0)	44.0 (11.0)	0.008
Albumin in g/dL, mean (SD)	3.97 (0.30)	3.96 (0.32)	3.98 (0.30)	4.0 (0.31)	0.288
Antihypertensive drugs, n (%)	171(52.9)	244(63.5)	453(61.4)	418(58.4)	0.020
Statins, n (%)	57(17.6)	82(21.4)	139(18.8)	150(20.9)	0.465
eGFR in ml/min/1.73 m^2^, mean (SD)	72.0 (18.2)	73.8 (18.8)	78.1 (18.1)	78.4 (18.0)	<0.001
eGFR, n (%)					
<60 ml/min/1.73 m^2^	85(26.3)	89(23.2)	123(16.7)	117(16.3)	<0.001
60–89 ml/min/1.73 m^2^	182(56.3)	209(54.4)	414(56.1)	401(56.0)	
≥90 ml/min/1.73 m^2^	56(17.3)	86(22.4)	201(27.2)	198(27.7)	

Abbreviation: SD: standard deviation; LDL-C: LDL-cholesterol; HDL-C: HDL-cholesterol; BMI: Body mass index; CVD: Cardiovascular diseases; IQR: interquartile range; eGFR: estimated glomerular filtration rate on the serum creatinine and cystatin C level.

^a^. *P* values for categorical variables were from Chi-square tests and for continuous variables were from ANOVA or Kruskal-Wallis Test where appropriate.

Poor olfaction was associated with poor kidney function at year 3. Compared with participants of good olfaction, the eGFR was on average 2.68 ml/min/1.73m^2^ lower (95%CI: -5.01, -0.35) among those with anosmia and 2.17 lower (95% CI: -4.32, -0.01) among those with hyposmia, after adjusting for demographic and lifestyle covariates (Table 2). The association was linear with a *P* for trend <0.001. These estimates were only modestly changed with further adjustment for overall health status, comorbidities, and use of antihypertensives and statins. Specifically, participants with anosmia had on average 3.0 ml/min/1.73 lower (-5.25, -0.75) eGFR than those with good olfaction, and the other two groups did not show significant difference in eGFR from the reference group. The associations were attenuated when we limited the analysis to participants with normal kidney function at year 3, but the difference remains statistically significant between the anosmia and good olfaction groups.

**Table 2:** Association of olfactory function with eGFR at baseline.

	N.	Mean eGFR, ml/min per 1.73 m^2^ (S.D.)	Difference in eGFR across olfaction groups (95% CI)			
			Model 1^a^	*P*	Model 2^b^	*P*
All participants, n = 2161						
Olfactory function						
Anosmia	323	72.0 (18.2)	-2.68 (-5.01, -0.35)	0.024	-3.00 (-5.25, -0.75)	0.009
Hyposmia	384	73.8 (18.8)	-2.17 (-4.32, -0.01)	0.049	-1.87 (-3.94, 0.21)	0.078
Moderate	738	78.1 (18.1)	0.94 (-0.82, 2.71)	0.295	1.15 (-0.55, 2.85)	0.186
Good	716	78.4 (18.0)	Reference		Reference	
			Trend *P* < 0.001		Trend *P* < 0.001	
Participants with baseline eGFR ≥ 60 ml/min per 1.73 m^2^, n = 1747						
Olfactory function						
Anosmia	238	80.2 (12.7)	-2.38 (-4.29, -0.46)	0.002	-2.35 (-4.25, -0.45)	0.015
Hyposmia	295	81.7 (12.7)	-1.26 (-3.00, 0.49)	0.156	-1.18 (-2.89, 0.53)	0.177
Moderate	615	83.9 (13.0)	0.36 (-1.03, 1.75)	0.615	0.54 (-0.84, 1.91)	0.444
Good	599	84.1 (13.1)	Reference		Reference	
			Trend *P* = 0.001		Trend *P* = 0.003	

Abbreviations: eGFR: estimated glomerular filtration rate on the serum creatinine and cystatin C level; S.D.: standard deviation; 95% CI: 95% confidence interval.

^a^ adjusted for age, gender, race, research site, education, smoking status, alcohol consumption, brisk walking

^b^ further adjusted for self-reported general health status, BMI, diabetes, hypertension, cardiovascular diseases, chronic lung diseases, LDL-cholesterol, log-transformed HDL-cholesterol, albumin, antihypertensive drugs, and statins uses.

In the analyses examining the relationship between olfaction at year 3 and kidney function at year 10, anosmia at baseline was associated with significant lower eGFR 7 years later (-5.31, 95%CI: -8.58, -2.04, *P* for linear trend = 0.002) compared to those with good olfaction (Table 3). This difference was however substantially attenuated and became statistically non-significant after further adjusting for baseline eGFR (between-group difference: -2.37, 95% CI: -4.91, 0.16, *P* for linear trend = 0.147). Kidney function was not different between other olfaction groups and those with good olfaction. The results were similar in the analyses limited to participants with normal kidney function at year 3.

**Table 3:** Association of baseline olfactory function with eGFR 7 years later.

	N.	Mean eGFR at yr 7, ml/min per 1.73 m^2^ (S.D.)	Difference in eGFR across olfaction groups (95% CI)			
			Model 1^a^	*P*	Model 2^b^	*P*
All participants, n = 1208^c^						
Olfactory function						
Anosmia	130	59.9 (18.6)	-5.31 (-8.58, -2.04)	0.001	-2.37 (-4.91, 0.16)	0.067
Hyposmia	194	63.4 (18.1)	-1.67 (-4.48, 1.14)	0.243	-0.25 (-2.43, 1.92)	0.819
Moderate	423	64.4 (18.4)	-1.38 (-3.57, 0.81)	0.218	-1.66 (-3.36, 0.03)	0.054
Good	461	67.1 (17.6)	Reference		Reference	
			Trend *P* = 0.002		Trend *P* = 0.147	
Participants with baseline eGFR ≥ 60 ml/min per 1.73 m^2^, n = 1033^d^						
Olfactory function						
Anosmia	102	65.0 (16.4)	-3.47 (-6.83, -0.11)	0.043	-1.77 (-4.58, 1.04)	0.217
Hyposmia	159	67.9 (15.7)	-1.38 (-4.23, 1.47)	0.342	-0.72 (-3.10, 1.65)	0.550
Moderate	366	67.6 (16.9)	-1.59 (-3.77, 0.59)	0.153	-1.67 (-3.49, 0.15)	0.072
Good	406	70.0 (16.0)	Reference		Reference	
			Trend *P* = 0.051		Trend *P* = 0.262	

Abbreviations: eGFR: estimated glomerular filtration rate on the serum creatinine and cystatin C level; S.D.: standard deviation; 95% CI: 95% confidence interval.

^a^ adjusted for age, gender, race, research site, education, BMI, smoking status, alcohol consumption, brisk walking, self-reported general health status, diabetes, hypertension, cardiovascular diseases, chronic lung diseases, LDL-cholesterol, log-transformed HDL-cholesterol, albumin, antihypertensive drugs and statin uses.

^b^ Further adjusted for eGFR at baseline.

^c^ Linear model with inverse probability weighting based on 1693 participants who were not dead before the follow-up of year 7.

^d^ Linear model with inverse probability weighting based on 1427 participants who were not dead before the follow-up of year 7.

In the analysis of olfaction and incident CKD between years 3 and 10, the OR was 1.23 (95%CI: 0.71, 1.24) comparing participants with anosmia to those with good olfaction (Table 4). In the analysis of olfactory function with subsequent CKD hospitalization, the CIR was 0.91 (95%CI: 0.64–1.29) comparing participants with anosmia to those with good olfactory function (Table 5). This analysis accounted for the competing risk of death, which was associated with poor olfaction as expected.

**Table 4:** Olfactory function in relation to incident CKD during 7 years of follow-up^a^ (n = 1033).

Olfactory function	N. incident cases / total (%)	Odds ratio (95% confidence interval)			
		Model 1^b^	*P*	Model 2^c^	*P*
Anosmia	37/ 102 (36.3)	1.36 (0.80, 2.30)	0.260	1.23 (0.71, 2.14)	0.458
Hyposmia	46/159 (28.9)	0.96 (0.61, 1.51)	0.853	0.92 (0.56, 1.51)	0.751
Moderate	121/366 (33.1)	1.21 (0.87, 1.69)	0.260	1.25 (0.88, 1.79)	0.213
Good	111/406 (27.3)	Reference		Reference	

Abbreviation: CKD: chronic kidney disease.

^a^ logistic model with inverse probability weighting based on 1427 participants who were not dead before the follow-up of year 7.

^b^ adjusted for age, gender, race, research site, education, BMI, smoking status, alcohol consumption, brisk walking, self-reported general health status, diabetes, hypertension, cardiovascular diseases, chronic lung diseases, LDL-cholesterol, log-transformed HDL-cholesterol, albumin, antihypertensive drugs and statin uses.

^c^ Further adjusted for eGFR at baseline.

**Table 5:** Olfactory function in relation to CKD hospitalization during 12 years of follow-up (n = 2531).

Olfactory	Censored	CKD hospitalization			Death without CKD hospitalization		
function		Event	CIR (95% CI) ^a^	*P*	Event	CIR (95% CI) ^a^	*P*
Anosmia	95	44	0.91 (0.64, 1.29)	0.583	226	1.54 (1.36, 1.74)	<0.001
Hyposmia	173	64	0.96 (0.70, 1.34)	0.828	220	1.24 (1.09, 1.42)	0.001
Moderate	404	118	0.95 (0.72, 1.25)	0.719	343	1.08 (0.96, 1.22)	0.218
Good	453	110	Reference		281	Reference	

Abbreviations: CKD: chronic kidney disease; CIR: cumulative incidence ratio; 95% CI: 95% confidence interval.

^a^ adjusted for age, gender, race, research site, education, BMI, smoking status, alcohol consumption, brisk walking, self-reported general health status, diabetes, hypertension, cardiovascular diseases, chronic lung diseases, LDL-cholesterol, log-transformed HDL-cholesterol, albumin, antihypertensive drugs and statin uses.

## Discussion

To the best of our knowledge, this is the first community-based study to examine poor olfaction in relation to kidney function and CKD risk among older adults. We found that anosmia was associated with current and future poor kidney function in these community-dwelling adults >70 years old, but not with incident CKD or future CKD hospitalization.

The kidney is the biological portal that filters out metabolic waste (e.g., uremic toxins) and extra water, maintaining the balance of water, minerals, and other metabolites in the blood. Human kidney function may begin to decrease with age, likely starting mid-to-late adulthood [44–46]. Such age-dependent decline in renal function may further exacerbate the kidney’s vulnerability to damages from accumulating internal and external toxins, forming a vicious cycle of accelerated renal dysfunction which may eventually lead to CKD [47]. CKD represents a substantial public health burden across the world. In 2017 alone, it was estimated that CKD had resulted in 35.8 million disability-adjusted life-years (DALYs) and 1.2 million deaths worldwide [48]. Further, a small portion of CKD patients will develop into ESRD, who will depend on dialysis for survival [49]. Besides, many more CKD patients suffer from other comorbidities (e.g., diabetes, CVD), further hastening premature death [2]. As CKD is potentially preventable, there is a need to find non-invasive, reliable, and easily assessable markers to monitor changes in kidney function over time.

Theoretically, poor olfaction could be a candidate marker as the olfactory system is sensitive to the toxic effects of accumulated uremic waste [50]. Unlike most other adult neurons in mammals, the olfactory system critically depends on neurogenesis to maintain normal olfactory function. In the peripheral olfactory system, the sensory receptor neurons originating from the olfactory epithelium renew every 6–8 weeks estimated in rodents [51]. In the central olfactory system, the neural progenitor cells in the subventricular zone provide interneurons to the olfactory bulb [52]. Excess uremic toxins may jeopardize these neural progenitor cells and inhibit their functions, possibly leading to impaired cell renewal/supplement and poor olfaction [50]. If this process is sensitive to changes in uremic toxins in the early stages of CKD, poor olfaction may be an early marker for future CKD.

While we are not aware of any study that has directly examined this possibility, several small hospital-based studies reported that patients with ESRD, renal failure or CKD had worse odor identification than controls [8–12]. For example, by comparing 136 CKD patients with 25 controls, Nigwekar, et al. reported that kidney disease was associated with 3.8 higher odds of poor odor identification after adjusting for potential confounding [12]. Although these studies support the accumulation of uremic toxins and olfactory impairment in patients with advanced CKD, they did not address the question of whether this could also occur in the earlier stages of the disease.

Compared to previous studies, this study is larger and includes relatively healthy community-dwelling older adults. Further, we aimed to examine whether olfaction is associated with renal function and predicts future risk of CKD in older adults. We found that anosmia or the total loss of the sense of smell was associated with both current and future poor kidney function, even when limited to participants who had normal kidney function at baseline. Further, the association appears to be monotonic across various levels of olfaction status. We adjusted for a range of potential confounders in the analyses, and the results strongly support an association between olfaction and kidney function. However, in our analyses for CKD risk, we did not find any significant associations of anosmia with either the risk of incident CKD or with CKD hospitalization. Taken together, despite the association of poor olfaction with decreased kidney function, it is unlikely to be an early marker of CKD in older adults after age 70.

Strengths of the current study include the relatively large sample size, community-based design, and comprehensive analyses on both kidney function and CKD risk after accounting for various potential confounders. The study also has multiple limitations. First, our study participants were relatively healthy but fairly old at enrollment, thus the results may not be readily generalizable to relatively young older adults or older adults with poor health. Our preliminary findings should lead to such investigations in younger older adults. Second, we only had two eGFR measures seven years apart, therefore our outcome measures are subject to misclassifications and biases due to non-participation, despite our controlling for loss-to-follow-up. Third, as in any observational study, we cannot exclude the possibility of residual confounding. Nevertheless, we have adjusted for a range of covariates in the analysis, including demographics, lifestyle, general health, comorbidities, and medications. Finally, olfaction was only measured at one timepoint, so we did not assess the possibility of using repeated measures of olfaction status to monitor changes in renal function and risk of CKD.

## Conclusions

In conclusion, in this community-based prospective cohort study of older adults > age 70, we found poor olfaction was associated with poorer kidney function but not the risk of CKD or CKD hospitalization. Future studies should investigate these associations in relatively younger adults.

## Supporting information

S1 Fig Analytic samples sizes for various analyses. (DOCX)
