# Supplementary figures and images for: Olfaction and kidney function in community-dwelling older adults
Source: PLoS One. 2022 Feb 25;17(2):e0264448. doi: 10.1371/journal.pone.0264448 (PMC8880852; doi:10.1371/journal.pone.0264448)

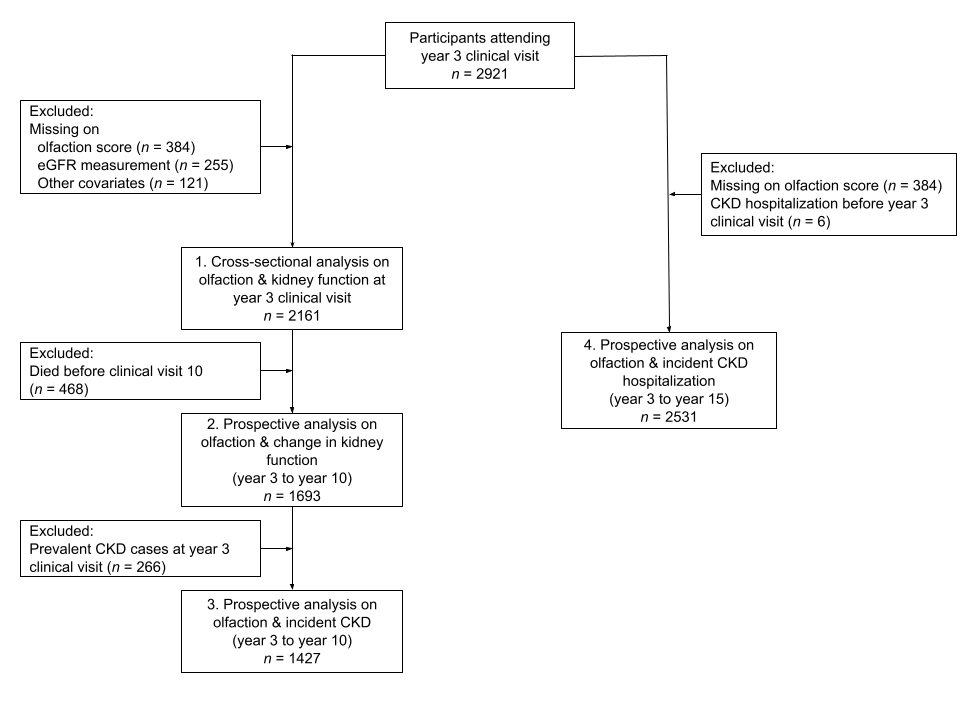


S1 Fig. Analytic samples sizes for various analyses

Supplement: S1 Fig — (DOCX) [file pone.0264448.s001.docx]
